# Complementary Role of CD4+ T Cells in Response to Pneumococcal Polysaccharide Vaccines in Humans

**DOI:** 10.3390/vaccines7010018

**Published:** 2019-02-11

**Authors:** Vibha Jha, Edward N. Janoff

**Affiliations:** 1 Mucosal and Vaccine Research Program Colorado (MAVRC), Infectious Diseases, University of Colorado Denver, Aurora, CO 80045, USA; vibha.jha@ucdenver.edu; 2 Eastern Colorado Rocky Mountain Veterans Affairs Medical Center, Denver, CO 80045, USA

**Keywords:** T-independent antigens, immunological responses, pneumococcal capsular polysaccharide, polysaccharide conjugate vaccine, antibody response, memory B cells, CD4+ T cells, carbohydrate-specific T cells

## Abstract

Bacterial pathogens expressing capsular polysaccharides are common causes of mucosal infections (pneumonia, intestinal), as well as often fatal, invasive infections (meningitis, bloodstream infections) in children and adults worldwide. These chemically simple but structurally complex carbohydrate structures on the bacterial surface confer resistance to recognition and clearance by the immune system through a range of mechanisms. Such recognition of capsular polysaccharides may be reduced by their limited ability to directly stimulate B cells and the T cells that may facilitate these humoral responses. The capsules may promote the evasion of complement deposition and activation and may sterically shield the recognition of other subjacent protein antigens by innate factors. Antibodies to capsular polysaccharides, elicited by infection and vaccines, may overcome these obstacles and facilitate bacterial agglutination at mucosal surfaces, as well as the opsonization and clearance of these organisms in tissues and the systemic compartment. However, the immunogenicity of these antigens may be limited by their lack of direct recognition by T cells (“T-independent” antigens) and their restricted ability to generate effective memory responses. In this review, we consider the mechanisms by which polysaccharides may initiate B cell responses and specific antibody responses and the role of T cells, particularly CD4+ follicular helper (TFH) cells to support this process. In addition, we also consider more recent counterintuitive data that capsular polysaccharides themselves may bind major histocompatibility antigen HLA class II to provide a more physiologic mechanism of T cell enhancement of B cell responses to capsular polysaccharides. Defining the contributions of T cells in the generation of effective humoral responses to the capsular polysaccharides will have important implications for understanding and translating this immunobiology for the development of more effective vaccines, to prevent the morbidity and mortality associated with these common mucosal and invasive pathogens in populations at risk.

## 1. Introduction

Polysaccharide capsules decorate the surfaces of a range of bacterial pathogens that are prominent causes of mucosal infections (e.g., pneumonia, otitis media, and sinusitis) which may also invade into the bloodstream (Table 1). *Streptococcus pneumoniae*, *Haemophilus influenzae*, and *Neisseria meningitidis* infections begin with asymptomatic upper respiratory tract colonization which can progress to pneumonia in the lower respiratory tract and represent the most common etiologies of meningitis. Maternal vaginal colonization with Group B streptococci predisposes to serious bloodstream infections and to meningitis in neonates. Although disease caused by *Salmonella enterica* serovar Typhi (*S. Typhi*) begins as an intestinal infection, the Vi bacterial capsule supports other virulence factors to subvert the local host defenses and produce prolonged bacteremia, particularly in children worldwide [1,2]. Two other encapsulated pathogens also originate in the intestine; *Klebsiella pneumoniae* infections are increasingly prevalent hospital-acquired infections and can be resistant to many or most antibiotics [3], while *Bacteroides fragilis* is most commonly associated with abdominal abscesses, although its capsule may also have distinct immunomodulatory properties [4,5,6]. Other encapsulated bacteria causing disease in humans are *Escherichia coli*, *Acinetobacter baumanii*, and *Staphylococcus aureus*, as well as the *Cryptococcus* species of yeast. Ultimately, encapsulated pathogens are responsible for tremendous numbers of lower respiratory, central nervous system, and both mucosal and invasive systemic infections which result in a high number of deaths in infants and children particularly in developing countries, as well as in older and immunocompromised adults worldwide [2,7]. 

As suggested above, bacterial polysaccharide capsules display a range of immunomodulatory effects, the majority of which are directed to limit the clearance of the organism. For example, the capsules of *S. pneumoniae* limit its adherence to respiratory epithelial cells, whereas those of *S.* Typhi and Group B *Streptococcus* facilitate the adherence and invasion of intestinal and cervical epithelial cells, respectively [1,10,23]. The Vi capsule of *S.* Typhi may inhibit both B and T cell responses, and that of *B. fragilis* has immuno-inhibitory activity. Generally, the common immuno-evasive effects of bacterial capsular polysaccharides include their ability to sterically decrease direct binding and the effects of innate antimicrobial peptides at mucosal surfaces, to abrogate direct activation of complement on the bacterial surface, to limit the inflammatory response, and to impair phagocytosis.

Despite their attenuated immunogenicity, bacterial polysaccharides are immune-reactive and were identified as the first non-protein antigens in the 1920′s by Avery and Heidelberger. Vaccines available against four of these encapsulated pathogens (*H. influenzae* type B, *S. pneumoniae*, *N. meningitidis*, and *S.* Typhi) are composed of their respective capsular polysaccharides either in pure form or conjugated to protein adjuvants (e.g., diphtheria toxoid, CRM197 (a single amino acid mutant of DT), tetanus toxoid). A live attenuated vaccine is also available for *S.* Typhi (Table 1). Vaccines containing protein adjuvants, first successfully developed against *H. influenzae* type B (Hib) in 1987, have been very successful in preventing disease caused by encapsulated pathogens, particularly in infants. Among these encapsulated bacteria, *S. pneumoniae* is one of the most common pathogens, accounting for over 1.0 million deaths worldwide annually [8]. This review focuses on understanding the role of pneumococcal polysaccharides as a prototype for understanding the immunobiology of responses to bacterial polysaccharides in general and vaccines against *S. pneumoniae* in particular.

## 2. Pneumococcal Polysaccharide Capsules (PPSs)

Pneumococcal polysaccharide capsules (PPSs) are the most critical virulence factor for *S. pneumoniae* [24]. The two major groups of serotypes of PPS differ in their synthesis and the enzymes involved in the exportation and covalent attachment of the PPS to the cell wall [25,26]. The first group, which contains all serotypes except type 3 and 37, follow a Wzy-dependent synthesis pattern whereby three different enzymes are responsible for the initiation of synthesis, polymerization, and exportation of the PPS. The second group includes type 3 and 37 and makes a single synthase enzyme that catalyzes all three steps of PPS synthesis [26]. PPS serotypes that follow a Wzy-pattern usually contain repeating units of three or four monosaccharides such that the type of monosaccharides, their sequence, and the differences in glycosidic linkages control differences in serological properties [26]. Types 3 and 37 are either made up of one (type 3, also among one of the least immunogenic serotypes) or two sugars (type 37) with structures simpler than those of other serotypes [26].

Although the infectivity and immunogenicity of a serotype do not appear to be dependent on these structural differences, the ionic nature of the PPS may dictate its immunogenicity. Biochemically, most PPSs are either neutral or negatively-charged, due to the presence of negatively-charged sialic acid groups [27]. However, the arrangement of positive and negative charges across the PPSs may regulate their immunogenicity. Zwitterionic PPSs (ZPSs; e.g., type 1) have alternating positive and negative charges which may enhance their immunogenicity by T cell-dependent mechanisms compared with non-ZPSs (non-Zwitterionic PPSs) [28,29,30]. Although capsular polysaccharides are composed of repetitive simple sugars, the quality (or binding affinity) of an antibody recognizing non-ZPSs, such as PPS-14, is directly proportional to the length of polysaccharide fragments, suggesting that the capsules actually represent complex conformational epitopes [31]. 

The thickness and complexity of PPSs may dictate the prevalence of a serotype to cause infection. The serotypes that are heavily encapsulated are more resistant to neutrophil-mediated killing and have a higher nasopharyngeal carriage prevalence when compared to their counterparts that carry less capsule [32]. Furthermore, among the serotypes that were not part of the PCV-7 vaccine (e.g., type 19F and type 15), those that were heavily encapsulated had shown a significantly higher probability to cause infection in the vaccinated population [32]. Thus, the ionic nature and thickness of PPSs control the immunogenicity and may affect the infectivity of a pneumococcal serotype. 

## 3. PPS-Specific Antibody Response and Memory B Cells

### 3.1. T Cell-Independent Antibody Production

Most antigens (antibody-generating molecules) require T cells to elicit humoral responses. The T-dependent (TD) antigens are processed by antigen-presenting cells (APCs) and the major histocompatibility complex-II (MHC-II)-peptide complexes bind to the T cell receptor of CD4+ T cells that provide cognate and soluble help to B cells (Table 2). In contrast, capsular polysaccharides are polymers consisting of repetitive sugar units, which classically renders them incompatible to processing by antigen presenting cells (APCs) and unable to be recognized by T cells. These antigens are known as T cell-independent (TI) antigens [33,34]. TI antigens are further classified into types 1 (TI-1) and 2 (TI-2). TI-1 antigens, such as lipopolysaccharides in mice, are ligands that engage the innate pattern recognition of toll-like receptors (TLRs), are mitogenic to B cells, and induce the production of non-stimulus-specific polyclonal antibodies, largely of the IgM class. These TI-1 ligands can stimulate antibody production by using neonatal B cells, mature B cells, and B cells carrying the X-linked immunodeficient gene (xid) (Table 2). TI-2 antigens, such as PPSs, bind to surface B cell receptors (BCRs) and generate antigen-specific antibodies only from mature B cells but not from the B cells of neonates or those carrying the xid mutation [33,35].

For TI-2 antigens, the membrane-bound pentameric IgM BCR on B cells binds 10–20 repetitive epitopes on the PPS, resulting in the clustering or cross-linking of the BCR; the binding of fewer epitopes does not elicit such activation [33]. Such binding activates Bruton’s tyrosine kinase (Btk) and induces the intracellular influx of ionized calcium, ultimately resulting in the activation, proliferation, and differentiation of B cells to produce PPS-specific antibodies [33]. The requirement of BCR crosslinking and Btk activation in B cells to produce polysaccharide-specific antibodies is supported by earlier studies in mice with severe combined immunodeficiency syndrome (SCID) and mice with mutations in the Btk gene that are unable to elicit an antibody response to polysaccharides [36,37]. Other complimentary mechanisms may assist in the enhancement of the TI PPS-specific antibody response, such as activation of the complement pathway via complement receptor 2 (CR2/CD21), cytokines produced by the activation of NK (Natural Killer) cells, and cytokines produced by activated B cells themselves [33,35]. CR2 is the receptor for the C3 complement fragmentation product C3d and involves the differentiation and growth of B cells [38,39]. Mice treated with cobra venom factor, which depletes C3, exhibit impaired immune responses to sub-optimal doses of TI-2 antigens.

In humans, C3-deficient subjects experience increased susceptibility to infections by encapsulated bacteria [40]. Neonatal and adult peripheral blood mononuclear cells (PBMCs) produced higher amounts of PPS-type 4-specific antibodies when they were co-stimulated with activating antibodies to CR2/CD21 [41]. Thus, the associated risk for infection with complement deficiency results from both impaired antibody responses and complement-mediated bacterial opsonization and lysis. Earlier reports suggest that a direct interaction between NK cells and B cells that results in the production of IFN-γ and the enhancement of polysaccharide-specific antibodies exists [42]. Interferon-gamma is involved in the class switch recombination process to produce IgG2, which is the predominant isotype of PPS-specific antibodies in adults [43]. Thus, T-independent type 2 responses to capsular polysaccharides may not require T cells, but other immune cells, such as NK cells, may contribute to the ability of B cells to respond to PPSs.

### 3.2. Nature of PPS-Specific Antibodies

Most adults produce low levels of PPS-specific antibody, typically of the IgG isotype and to a lesser extent, of the IgA isotype, in the absence of prior immunization. Such antibodies may have been generated by prior exposure to colonization or infection with pneumococcal strains, to cross-reacting organisms, such as certain strains of *H. influenzae* and *E. coli*, or as polyreactive antibodies that are not directed specifically to the PPS alone [44,45]. These baseline antibodies and PPS-specific antibodies, in response to symptomatic infection and vaccination in adults, are usually of the IgG2 subclass [45]. In contrast, the ability of young children under two years of age to produce antibodies of the IgG1 subclass, but their limited ability to produce the IgG2 subclass, may result in their predisposition to pneumococcal pneumonia, bacteremia, and meningitis, as well as in their compromised ability to respond to the pure pneumococcal polysaccharide vaccine (earlier vaccine PPSV-14, now PPSV-23). With the advent of the newer protein-polysaccharide conjugate vaccines (initially PCV-7, now PCV-13), young infants generate robust responses to the PPS of the IgG1 subclass, responses that provide more effective protection against invasive pneumococcal disease [46,47]. Furthermore, the limited diversity and avidity of PPS-specific antibodies may affect their opsonophagocytic characteristics [48,49]. Adults vaccinated with the pure polysaccharide pneumococcal vaccine PPSV-23 produce oligoclonal PPS-specific IgG antibodies. These specific antibodies, as described with those specific to types 6B, 14, and 23F that are constrained in their diversity, show functional activity to opsonize *S. pneumoniae* bacteria [49].

### 3.3. Memory B Cell (mB) Responses to the PPS

A controversial area in our understanding of the responses to PPSs is the ability of these repetitive antigens to elicit immunological memory [50]. Memory response is defined as the ability of the adaptive immune system to generate more rapid and higher levels of antigen-specific antibodies in response to a secondary antigenic challenge when compared with the primary challenge. Such recall responses to “booster” doses, most consistently observed with T-dependent antigens (e.g., diphtheria or tetanus toxoid proteins), are typically derived from previously generated memory B cells that produce antibodies of the IgG and IgA isotypes and, to a lesser extent, IgM isotypes. These antibodies forming the memory response display higher avidity for the antigen and are functionally superior in their ability to neutralize the pathogen [51]. Antigens with repetitive structures like PPSs may stimulate B cells directly, as above, by crosslinking the surface B cell receptor that recognizes it and producing limited amounts of IgM, whereas IgG and memory responses are limited [52,53]. In this context, the responses to revaccination with TI antigens are not greater than those to primary immunization [54]. The introduction of a protein carrier linked to the PPS in conjugate vaccines such as PCV-13 (and conjugate vaccines against the capsular polysaccharide of *H. influenzae* and *N. meningitidis*) has resulted in capsule-specific antibodies of greater magnitude and more recall memory with substantial clinical efficacy in young children. These effects are most likely derived from the ability of the carrier protein to engage CD4+ T cells specific for the protein, but not the polysaccharide, that provides both soluble and cognate signals to adjacent capsule-specific B cells [53]. 

The generation and persistence of memory B (mB) cells are essential for the production of long-lasting antigen-specific antibodies which can then prevent recurrent infections. Phenotypically, human mB cells largely express the surface marker CD27+, most often class switched (e.g., IgM−, IgG+, or IgA+), but also non-class switched (IgM+). In children, the increasing frequencies of mB cells with age are inversely proportional to the prevalence of *S. pneumoniae* infections. Additionally, the lower numbers of mB cells in older adults may contribute in part to their increased incidence of pneumococcal disease [55,56]. These correlations suggest a role for mB cells in mounting a successful immunological defense against *S. pneumoniae*. Evidence for the generation of mB cells, in response to the PPS in adults, is derived from the presence of PPS-specific B cells in adults vaccinated with the pure pneumococcal polysaccharide vaccine PPSV-23 [57]. Westerink and colleagues labeled PPS type 14 and PPS type 23F to track PPS-specific CD27+ IgM+ or CD27+ IgM− B cells in adults immunized with PPSV-23. The majority of PPS-specific mB cells before vaccination were naïve (CD27-IgM+). Post-vaccination, percentages of the class-switched PPS-specific mB cells (CD27+, IgM−) remained the same, whereas percentages of non-class switched PPS-specific mB cells (CD27+ IgM+) increased significantly [57]. Although providing direct evidence for the existence of PPS-specific mB cells, the long-term survival of these mB cells and their functional role in maintaining circulating antibody levels and the prevention of future infections has not been determined.

Both healthy adults and infants generate mB cells specific to the PPS in response to the PPS-conjugate vaccine, PCV-7. Primary and memory immune responses to the PPS-protein conjugated vaccine PCV-7 were investigated in adults and in infants less than 12 months old. The primary PPS-specific antibody responses were higher in adults compared to the infants, who mostly produced antibodies of the IgM isotype. The infants required a secondary booster dose of PCV-7 to induce PPS-specific mB responses, whereas adults did not need a booster dose to mount a PPS-specific mB response [58]. Another clinical study conducted in older adults (≥70 years) identified differences in primary and secondary immune responses induced by PPSV-23 and PCV-7. Primary immunization with PPSV-23 induced lower PPS-specific IgG and a lower secondary response, even when the booster dose consisted of PCV-7. In contrast, both primary and secondary PPS-specific responses were more robust when older adults were immunized with PCV- 7 and boosted with a second dose of PCV-7 [59]. The enhanced immunogenicity of the conjugate vaccine was much more striking in children than in adults, who may well have had earlier exposure to pneumococcal antigens than young infants. Indeed, as noted earlier, the antibodies generated following asymptomatic colonization with *S. pneumoniae* [44,45] suggest that these asymptomatic exposures may initiate B cell recognition and memory responses to the PPS.

In summary, the presence of a protein conjugated to polysaccharide in the vaccine directs attention to the role of APCs and T cells in enhancing the magnitude and the quality of PPS-specific memory responses in an at-risk population of older adults.

## 4. Collaborative T Cell-Dependent B Cell Responses

### 4.1. CD4+ T Cells Contribute to Effective Memory B Cell Responses

The differentiation of B cells in response to TI-2 antigens has been proposed to occur at extrafollicular sites [60,61,62]. However, B and T cells most often interact at the border of lymphoid follicles and a subset migrates inside the follicle and active germinal centers. In the germinal center (GC), antigen-activated B cells undergo somatic hypermutation (SHM) of the antigen-binding variable regions and class switch recombination (CSR) (e.g., IgM to IgG or IgA) of the effector constant regions. B cells are selected by increased BCR avidity and preferential engagement by the local CD4+ T follicular helper (TFH) cells. These selected B cells then differentiate to become either antibody-producing plasma cells or long-lived memory B cells [63]. Of the multiple T cell subsets that are derived from naïve CD4+ T cells, TFH cells are specialized subsets of T cells that most specifically and effectively interact with B cells to generate effective humoral responses. TFH cells utilize both soluble factors, such as IL-21 and IL-4, and cognate receptor-ligand interactions (e.g., ICOS-ICOSL, CD40L-CD40, CD28-CD80/CD86 on T and B cells, respectively) to effect SHM, CSR, and B cell differentiation [64,65].

### 4.2. T Follicular Helper (TFH) Cells and Their Role in the PPS-Specific Humoral Response

Currently, only a few studies explore the role of TFH cells in the generation of the anti-PPS response. The immunization of mice with mutant *S. pneumoniae* R614 expressing ovalbumin (OVA) resulted in increased percentages of TFH cells in the germinal centers, whereas injecting mice with clodronate, that depletes macrophages and monocytes, caused a 50% reduction in the TFH cell population [66]. In humans, the percentages of circulating TFH (cTFH) cells expressing inducible costimulatory molecule (ICOS), which sustains TFH cell function and phenotype, were recently reported to increase in the blood in response to the pure pneumococcal polysaccharide vaccine. Moreover, the levels of PPS-specific IgG correlated significantly with increases in ICOS+ cTFH cells, suggesting a contribution of TFH cells in the anti-PPS humoral response [67]. These studies indicate that polysaccharide vaccines may generate TFH cell responses in humans, with a link to a functional association with the immunogenicity of the vaccine.

Overall, T cell responses, particularly those of TFH cells, following polysaccharide vaccines may participate in the formation of B cell follicles and the subsequent production of high avidity antibodies produced by short- and long-lived plasma cells and memory B cells that serve as an inducible reservoir for plasma cells [68]. Such activity may vary based on the presentation of the capsular polysaccharide antigens in the context of vaccination with proteins or infection with whole bacteria [69].

### 4.3. Differential Requirements of CD4+ T Cells in Humoral Response to Whole Cell S. pneumoniae versus the Polysaccharide-Protein Conjugate Vaccine

PPSs are covalently attached to the thick layer of the cell wall which is made up of peptidoglycan, allowing the anchoring of various protein antigens of the intact *S. pneumoniae* organism. This architecture results in the induction of immune responses against the PPS that is complemented by the recognition of protein antigens that intercalate through the capsule, and of several TLR-ligands interspersed in the cell wall of and within bacteria, resulting in TI-2, T cell-dependent, and TI-1 responses, respectively. Mice immunized with heat-killed whole *S. pneumoniae* type 14 bacteria generated anti-PPS-type 14-specific IgM antibodies independent of T cells, whereas IgG responses specific to pneumococcal surface protein A (PspA) and PPS-type 14 were T cell-dependent with the requirement of the CD28 and CD40L costimulatory signals. However, the rise in serum PPS-specific IgG antibody levels after a booster dose were very low, suggesting a compromised memory response to the polysaccharide [70]. Moreover, different immunological pathways appeared to regulate the PPS-specific responses in mice immunized with either heat-killed *S. pneumoniae*-type 14 or soluble complexes containing PPS-type 14 conjugated to pneumococcal protein PspA (PPS-PspA). The PPS-specific response to killed whole cell bacteria was devoid of a memory response and was T cell-independent. In contrast, the immunization of mice with the PPS-PspA conjugate generated PPS- and PspA-specific IgG responses that were T cell-dependent, required costimulatory signals induced via ICOS, CD28, and CD40, and generated robust memory responses. Thus, the PPSs in particulate whole cell bacteria have characteristics similar to true TI-2 antigen, producing primarily IgM and limited memory. In contrast, PPSs in the form of a soluble complex of chemically-linked PPS-PspA have characteristics similar to a polysaccharide conjugate vaccine with a greater induction of IgG and memory responses. These later effects appear due to the activation of T cell-dependent signaling pathways, such as the activation of SLAM-associated proteins (SAP) that are essential for maintaining T and B cell interactions, the formation of healthy germinal centers, and the generation of immunological memory [71,72,73].

In humans, studies that clearly outline the differences in immunological responses between whole cell bacteria and polysaccharide conjugate vaccines are not available. However, humanized SCID mice were used to demonstrate the complementary role of CD4+ T cells in the production of PPS-specific antibody responses. Humanized SCID mice immunized with heat-killed type 3 *S. pneumoniae* required CD14+ monocytes, CD4+ T cells, and CD40-CD40L interaction for the generation of PPS-specific IgG and IgM responses [74]. Consistent with these results in mice, cultured human PBMCs stimulated with PPS-type 19F were unable to produce PPS-specific antibody-secreting cells in the presence of inhibitory anti-CD40L antibody, indicating the requisite role of CD40-CD40L interactions in the generation of PPS-specific humoral responses [75].

Thus, responses to TI-2 PPS antigens may involve both direct B cell activation as well as complementary help by CD4+ T cells, particularly TFH cells, in generating responses to pneumococcal polysaccharide conjugate vaccines such as PCV-7 and PCV13. In addition to their complimentary activity in facilitating anti-PPS responses, T cells have been recently proposed as having a more direct interaction with APCs that present the PPS in the context of MHC-II.

### 4.4. Direct Role of CD4+T Cells in Recognizing Capsular Polysaccharide 

As discussed in an earlier section (on Pneumococcal Polysaccharide Capsules), the distribution of positive and negative charges across the polysaccharide structure may regulate the immunogenicity of the PPS. In particular, zwitterionic polysaccharides (ZPSs) (e.g., PPS-type 1) are reported to be processed by APCs and presented to CD4+ T cells in the context of MHC-II, thus, enabling ZPSs to be more immunogenic than non-ZPSs [28,30]. Using human Raji B cell lines, Raji B cell lines lacking MHC-II, and iNOS-deficient mice, Velez et al. performed colocalization studies using confocal microscopy and immunoprecipitation to show that APCs use an oxidative-processing pathway to break PPS-type 1 into smaller fragments that colocalize with MHC-II to activate CD4+ T cells [28,29]. Unlike PPS-type 1, most PPSs are not ZPS. However, limited but growing evidence suggests that carbohydrate-specific T cells are activated by APCs, which process non-ZPSs in the presence of covalently-linked protein.

A non-zwitterionic capsular polysaccharide, type III of group B *Streptococcus* (GBSIII), was conjugated to an ovalbumin peptide (OVAp) to demonstrate that MHC-II on APCs was associated with carbohydrate (CHO) fragments and OVAp in a single complex. Binding of CHO to MHC-II on Raji B cell lines required the conjugation of GBS-III to OVAp (III-OVAp); this association was lost when Raji B cell lines were stimulated with GBS-III alone or GBS-III and unconjugated OVAp. Furthermore, the investigators were able to identify and purify CHO-specific CD4+ T cell clones from mice immunized with GBS-III-OVAp [27,76]. Similar experiments were done with the pneumococcal non-ZPS type 14 and type 9F. In this work, confocal microscopy was used to visualize the colocalization of MHC-II on the Raji B cell lines with PPS-type 14 or 9F only when the PPSs were conjugated to the diphtheria toxoid (cross-reactive material; CRM197 adjuvant protein (cross-reacting material; CRM197) used in vaccines such as PCV-7 and PCV-13) [77]. 

The involvement of CD4+ T cells has been studied in mice immunized with PPS-type 3 conjugated to proteins [78]. PPS-3 specific IgG was generated when mice were immunized with PPS-3 conjugated to keyhole limpet hemolysin (KLH; PPS-3-KLH) or to OVAp (PPS-3-OVAp) but not when they were immunized with PPS-3 alone or with unconjugated OVA or KLH. Additionally, there was a rapid rise in the serum levels of anti-PPS-type-3 IgG (hallmark of a memory response) only when mice were given the booster dose of either PPS-3-OVA or PSP-3-KLH but not when the booster dose contained the KLH or OVAp alone, suggesting that a PPS-specific memory response is independent of the type of conjugate protein used. PPS-3-specific CD4+ T cell hybridomas were generated from CD4+ T cells isolated from mice immunized with PPS-3-OVA [78]. These experiments in mice explain a mechanism by which protein conjugated to the PPS facilitates the processing and presentation of the PPS by APCs, resulting in the induction of CHO-specific CD4+ T cells which is not dependent on the type of protein used in the PPS-protein conjugated complex. These data also led to the hypothesis that activation of CHO-specific T cells might be driving the improved generation of PPS-specific IgG after the secondary immunization with PPS-protein conjugated vaccine in humans. Further investigation to detect the CHO-specific T cells in humans immunized with the PPS-conjugated protein vaccine is necessary to confirm the complementary role of PPS-specific CD4+ T cells in generating long-lasting PPS-specific immunological memory. 

## 5. Conclusions and Future Considerations

Bacteria expressing capsular polysaccharides represent a broad range of organisms causing a diversity of infections, almost all of which begin and largely cause disease at mucosal sites. *S. pneumoniae* is perhaps the most common infectious pathogen in this group and its immunobiology, including the large number of distinct serotypes, serves as a prototype for understanding the ability to and mechanisms of generating effective antibody responses to its capsules by T-independent and -dependent pathways. The repetitive sugar units of the PPS can engage and activate B cells through the surface B cell receptor (BCR) as classical TI-2 antigens with limited immunogenicity, particularly in infants, and evoking a limited memory B cell response in children and adults. However, distinct mechanisms of T cell-independent B cell engagement by BCR crosslinking, activation of Btk and the CR2/CD21 receptor on B cells, and the potential contribution of NK cell-derived IFN-γ-mediated CSR events support the feasibility and activity of TI-2 responses. 

The proteins covalently conjugated to the capsular polysaccharides engage protein-specific CD4+ T cells to provide the cognate and soluble help to the proximate polysaccharide-specific B cells. These complementary T cell-dependent activities, as observed with pneumococcal conjugate vaccines (e.g., PCV-13), have elicited substantially greater antibody responses and remarkable protection against invasive disease, particularly in infants. In addition to the recognition and exploitation of the observation that conjugated proteins can augment antibodies to capsular polysaccharides indirectly, recent novel findings suggest that selected capsular polysaccharides can engage CD4+ T cells directly and, specifically, in mice. As with the classic T cell-dependent responses to proteins, the protein-conjugated polysaccharides were processed by antigen-presenting cells and presented to CD4+ T cells by MHC-II, in combination with the processed protein adjuvants, resulting in the generation of polysaccharide-specific T cells. Thus, current research is directed towards understanding both the ability of polysaccharides to directly engage B cells and the indirect and direct effects of CD4+ T cells, particularly TFH cells. 

The growing recognition of the interdependent roles of B and T cells in the generation of effective antibody responses to capsular polysaccharides, heretofore considered “T cell-independent” antigens, allows more targeted approaches to developing effective vaccines to these prominent virulence factors. Other interactions that merit incorporation into this evolving paradigm includes the role of antigen presentation of capsular polysaccharides by dendritic cells, as well as the role of NK cells and the associated interferon production in effective humoral responses. The invocation of these cells and factors in initiating and enhancing capsule-specific antibody responses, as well as long-term memory through B cell recruitment and maintenance, may involve distinct applications in different populations at an increased risk for disease. These groups include very young children, older adults, and persons with underlying immune compromise, such as those with HIV-1 infection, each of whom may vary in the immune target that requires supplementation. We must begin by trying to understand the normal physiological process so that the pathological defects may be identified and overcome. The goal is to most efficiently and effectively generate protection, including mucosal protection, against these common and serious encapsulated pathogens in persons at risk.

## Figures and Tables

**Table 1:** Clinically important encapsulated bacteria and vaccines. PCV13, Pneumococcal polysaccharide conjugate vaccine 13; PPSV23, Pure pneumococcal polysaccharide vaccine 23. PRP-D, polyribosyl-ribitol-phosphate-Diphtheria toxoid (DT) conjugate vaccine; PRP-CRM, PRP-conjugate vaccine containing CRM197, a mutated DT protein; PRP-OMP, PRP conjugate vaccine containing meningococcal outer membrane protein; PRP-T, PRP conjugate vaccine containing tetanus toxoid. MPSV4, Tetravalent meningococcal polysaccharide vaccine; MCV4, tetravalent meningococcal polysaccharide conjugated with diphtheria toxoid or diphtheria CRM197 protein. Vaccines against *N. meningitidis* type B are directed to surface proteins rather than capsular polysaccharides. K antigens, surface exposed capsular polysaccharides in *K. pneumoniae.* Vi, Virulence antigen (shared capsular polysaccharides). A Vi-protein conjugate vaccine is in late-stage Phase 3 testing for efficacy, particularly in young children.

Name (Gram Character and Morphology)	No. of Capsular Serotypes	Type of Illness	Annual Mortality (Cases)	Polysaccharide Vaccines Available	Refs
*Streptococcus pneumoniae* (Gram-positive cocci)	94	Pneumonia, Otitis media, Meningitis	1.5 million (500,000 children ≤ 5 years of age)	PCV-13, PPSV-23	[8,9]
*Streptococcus agalactiae* (Gram-positive cocci)	9 (type 3 is predominant)	Neonatal sepsis, Meningitis, Pyrogenic infection	150,000 neonates	None currently licensed	[10]
*Haemophilus influenzae* (Gram-negative coccobacilli)	6 (a–f) (type b is predominant)	Pneumonia, Meningitis, Cellulitis, Arthritis	371,000, especially children ≤ 4 years of age	PRP-D, PRP-CRM, PRP-OMP, PRP-T	[11,12]
*Neisseria meningitidis* (Gram-negative cocci)	13 (5 types are predominant)	Meningitis, Pneumonia, Arthritis, Septicemia	15,000	MPSV4, MCV4 (types A, C, Y, and W-135)	[13,14]
*Klebsiella pneumoniae* (Gram-negative bacilli)	>78 K antigens (K2 and K1 are predominant)	Urinary tract infections, Pneumonia, Bacteremia	Not available	None currently licensed	[15,16,17,18]
*Salmonella enterica* serovar Typhi (Gram-negative bacilli)	1 (Vi)	Enteric fever, Gastrointestinal infection, Septicemia	150 to 210,000	Ty21a (Oral live attenuated vaccine) and Vi PS* (injectable vaccine)	[1,19,20,21,22]
*Bacteroides fragilis* (Gram-negative bacilli)	2	Abdominal abscess	Not available	None currently licensed	[4,5,6]

**Table 2:** The primary stimuli and receptors generating antibody responses. APC, antigen presenting cell; MHC-II, Major histocompatibility-II; LPS, lipopolysaccharide; TLR, Toll-like receptor.

Stimulus Class	Examples	Receptors	Antibodies Produced	Antibody Isotypes	Recall Memory Responses
T-dependent	APC-processed protein peptides with MHC-II	T cell receptor	Antigen-specific	IgM, IgG, IgA, IgE	Yes
T-independent type 1 (TI-1)		Innate	Polyclonal (not stimulus-specific)	IgM	No
	LPS (mice)	- TLR-4			
	CpG	- TLR-9			
	Poly-IC (dsRNA)	- TLR-3			
	R848	- TLR7/8			
T-independent type 2 (TI-2)	Capsular polysaccharides	B cell receptor	Antigen-specific	IgM, IgG, IgA	+/−
